# A Predictive Web-Based Nomogram for Elderly Patients Newly Diagnosed as Uveal Melanoma: A Population-Based Study

**DOI:** 10.3389/fmed.2022.799083

**Published:** 2022-06-23

**Authors:** Meng Lv, Xinhua Yan, Yuanxing Tu

**Affiliations:** Department of Ophthalmology, Lanxi People’s Hospital, Jinhua, China

**Keywords:** nomogram, elderly, uveal melanoma, overall survival, SEER

## Abstract

**Background:**

Uveal melanoma (UM) is the most frequent primary intraocular tumor in adults. This study aims to develop a nomogram and an individualized web-based calculator to predict the overall survival (OS) of elderly patients with UM.

**Methods:**

Patients aged more than 60 years and diagnosed with UM were derived from the Surveillance, Epidemiology, and End Results (SEER) database during 2004–2015. The selected patients were randomly divided into training and validation cohorts. In the training cohort, the univariate and multivariate Cox analyses were carried out to determine the independent prognostic factors, and the predictors were integrated to establish a nomogram for predicting the 1-, 2-, and 3-year OS of elderly patients with UM. The discrimination of the nomogram was validated by receiver operating characteristic (ROC) curves and the area under the curve (AUC). The clinical practicability and accuracy of the nomogram were evaluated by the calibration curves and decision curve analysis (DCA). A web-based survival calculator was then constructed using a fitted survival prediction model (https://yuexinupup.shinyapps.io/DynNomapp/).

**Results:**

A total of 1,427 patients with UM were included in this study. Age, T stage, N stage, M stage, marital status, sex, and radiotherapy (RT) were identified as independent prognostic factors. Based on the abovementioned factors, the nomogram was then constructed. The AUC values of the nomogram predicting 1-, 2-, and 3-year OS were 0.841, 0.801, and 0.768 in the training cohort, and 0.745, 0.717, and 0.710 in the validation cohort, respectively. The calibration curves and DCA also indicated the good performance of the predictive model.

**Conclusion:**

This study established and validated a novel nomogram risk stratification model and a web-based survival rate calculator that can dynamically predict the long-term OS for elderly patients with UM.

## Background

Uveal melanoma (UM) is the most prevalent primary intraocular malignancy in adults, accounting for 5% of all melanoma cases and mainly originating from the choroid (90%), followed by the ciliary body (7%) and the iris (2%) (1). Approximately 5–10 individuals per million suffer from UM worldwide each year (2). Over one-third of patients with UM will evolve into metastatic disease with a median survival of less than 1 year (3). It has been reported that UM is an age-associated disease and the incidence increases with age. Shields et al. confirmed that the incidence of UM was lower among younger children and teenagers (4, 5). Compared with other age groups, elderly patients with UM had significantly increased mortality, with a hazard ratio (HR) of 1.679 (6). Considering the aging trend of the population around the world, the number of elderly patients with UM is likely to constantly increase in the future. Studying the prognostic factors and a comprehensive prognostic model may provide some references for personalized medical decisions of elderly patients with UM (7, 8).

A number of clinical and histopathological features have been investigated in order to predict the prognosis for patients with UM; age and tumor size have been identified to be prognostic indicators for patients with UM (9). In addition, light iris color is one of the established risk factors for UM (10), and another study conducted by Lemke AJ et al. has examined the association between light iris color and the risk of death from UM (11, 12). Moreover, among the subtypes of UM, iris melanoma is associated with a better prognosis than ciliochoroidal melanoma (13). To the best of our knowledge, the prognostic study exclusive to elderly patients with UM has not been reported. With the integration of various important factors, a nomogram can individually quantify the probability of a certain clinical event of patients, including the survival rate or recurrence. Therefore, the nomogram has become a convenient clinical tool facilitating clinical decision-making and risk stratification (14). Although the nomogram can improve predictive accuracy, it may be difficult to use in clinical practice due to the need of performing manual calculation. The web-based calculator based on nomogram allows to enhance the accuracy and practicality of survival prediction based on the nomogram. This study aimed to develop a predictive nomogram and a web-based survival rate calculator that can dynamically predict the long-term overall survival (OS) for elderly patients with UM based on a population-based retrospective cohort study using the data from the Surveillance, Epidemiology, and End Results (SEER) database.

## Materials and Methods

### Study Population

**FIGURE 1:**
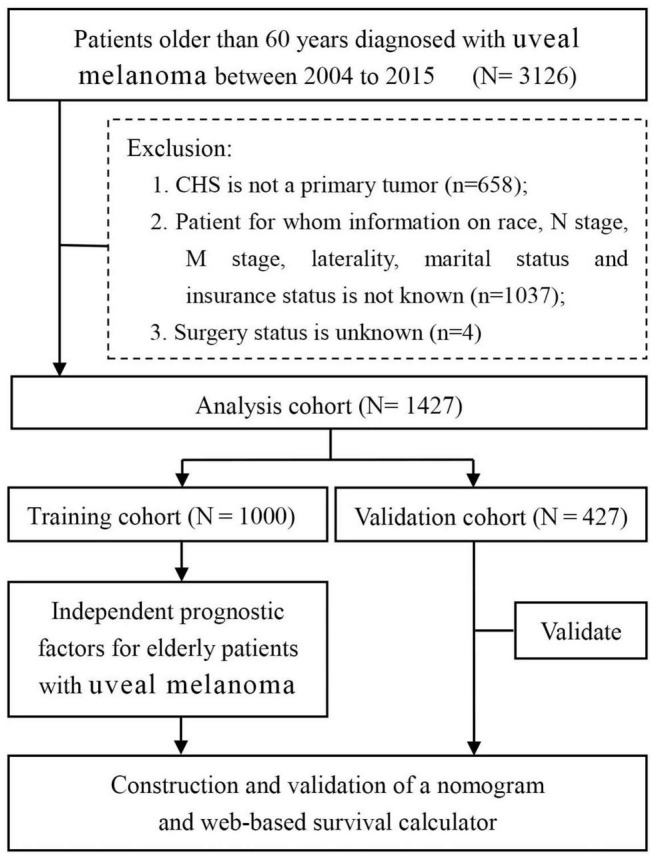
Flow diagram of filtering and selecting patient records from the SEER database. SEER: Surveillance, Epidemiology, and End Results.

The SEER database, supported by the National Cancer Institute, constituted approximately 27.8% of the US population. The research data of patients with UM were downloaded from the SEER database by the SEER*Stat software version 8.3.5 during 2004–2015. We used the International Classification of Disease for Oncology third edition (ICDO-3) to identify UM cases, namely, C69.3 (choroid) and C69.4 (ciliary body and iris). The inclusion criteria of patient selection were as follows: (a) patients were diagnosed with UM during 2004–2015; (b) patients older than 60 years; and (c) patients with complete follow-up. The exclusion criteria of patient selection were as follows: (a) patients whose selected variables were unknown; (b) UM was not the patient’s first primary tumor; and (c) patients were diagnosed with autopsies or death certificates. Selected variables included age, marital status, insurance status, sex, race, primary site, laterality, histologic type, T stage, N stage, M stage, surgery, radiotherapy (RT), chemotherapy, and historic stage. X-tile software provides the best cutoff point and changes continuous variables into categorical variables; therefore, the age of patients is divided into three groups, including <70 years, 70–82 years, and >82 years (15). The study endpoint was OS, which was defined as the time between the date of disease diagnosis and the date of death from any disease cause. Figure 1 shows the workflow for the study. Patients were pathologically categorized by the 7th edition of the TNM staging system of the International Union Against Cancer/American Joint Commission on Cancer (UICC/AJCC).

### Institutional Review Board Approval

This study was strictly abide by the Declaration of Helsinki and exempted from Institutional Review Board oversight from Lanxi People’s Hospital for the reason that patient information in the SEER program is de-identified and publicly available.

### Statistical Analysis

In our study, the statistical method was performed using SPSS version 25.0 and R software (version 3.5.1). Differences in baseline characteristics were examined using the chi-square test. Elderly patients with UM were randomized into training and validation cohorts at a ratio of 7:3. The univariate Cox regression analysis was first used to explore OS-related variables. Those variables with *p* < 0.05 were further analyzed by the multivariate analysis. Then, the variables with *p* < 0.05 in the multivariate Cox analysis were finally determined as the independent prognostic factors of elderly patients with UM. Later, we developed a prognostic nomogram for predicting the 1-, 2-, and 3-year OS. This study performed internal and external validation of the model and plotted the time-dependent receiver operating characteristic (ROC) curves, and the value of area under the curve (AUC) was used to estimate the performance of the nomogram. The calibration curves and decision curve analysis (DCA) curves were also generated. Besides, we calculated the total point of all patients based on nomogram and divided them into three subgroups at different risks of mortality according to an optimal cutoff value determined by X-tile, including high-, medium-, and low-risk groups. The Kaplan-Meier survival analysis with the log-rank tests was conducted to compare the survival difference among the three subgroups. The value of *p* < 0.05 was considered statistically significant.

## Results

### Baseline Data of Elderly Patients With Uveal Melanoma

In this study, we finally included 1,427 elderly patients diagnosed with UM. The study population were randomly allocated into two cohorts, namely, the training cohort (*n* = 1,000) and the validation cohort (*n* = 427). Patients in the age groups of <70 years (50.3%) and 70–82 years (38.7%) made up the majority of the study sample. The number of married patients (63.3%, 918 cases) was nearly twice than that of unmarried patients (35.7%, 509 cases). Almost all patients had insurance coverage (99.0%, 1,412). In the distribution of race, most of the patients (98.2%, 1,401 cases) were white. Both the male-to-female ratio and the right eye-to-left eye ratio were close to 1:1. The most common primary site of UM was choroid (89.7%, 1,280 cases), while another common site was ciliary body (10.3%, 147 cases). According to the 7th AJCC/TNM staging system, 1,124 cases were staged as T1–T2, while 303 cases were staged as T3–T4. In terms of the N and M stages, 1,398 cases were staged as N0, 29 cases as N1, 1,408 cases as M0, and another 19 cases as M1. In terms of treatment information, only a quarter of patients (25.7%) underwent surgery, while the other cases weren’t. Most of the patients received RT (1,092 cases, 76.5%). The detailed demographic and clinicopathological information of the two cohorts are listed in Table 1.

**TABLE 1:** Baseline demographic and clinicopathologic data of elderly patients with UM.

Variables	The total cohort	The training cohort	The validation cohort	*P-*value
	*N*, (%)	*N*, (%)	*N*, (%)	
**Age (years)**				0.367
60–69	717 (50.3)	511 (51.1)	206 (48.2)	
70–82	553 (38.7)	386 (38.6)	167 (39.1)	
83–98	157 (11.0)	103 (10.3)	54 (12.6)	
**Marital status**				0.603
Married	918 (63.3)	639 (63.9)	279 (65.3)	
Unmarried	509 (35.7)	361 (36.1)	148 (34.7)	
**Insurance status**				0.772
Insured	1412 (99.0)	990 (99.0)	422 (98.8)	
Uninsured	15 (1.1)	10 (10.0)	5 (1.2)	
**Sex**				0.109
Female	688 (48.2)	496 (49.6)	192 (45.0)	
Male	739 (51.8)	504 (50.4)	235 (55.0)	
**Race**				0.785
Black	10 (0.7)	8 (0.8)	2 (0.5)	
Other	16 (1.1)	11 (1.1)	5 (1.2)	
White	1401 (98.2)	981 (98.1)	420 (98.4)	
**Primary site**				0.702
Choroid	1280 (89.7)	899 (89.9)	381 (89.2)	
Ciliary body	147 (10.3)	101 (10.1)	46 (10.8)	
**Laterality**				0.065
Left	709 (49.7)	513 (51.3)	196 (46.0)	
Right	718 (50.3)	487 (48.7)	231 (54.0)	
**Histologic type**				0.103
Malignant melanoma	1155 (80.9)	822 (82.2)	333 (78.0)	
Spindle cell melanoma	117 (8.2)	79 (7.9)	38 (8.9)	
Epithelioid cell melanoma	50 (3.5)	36 (3.6)	14 (3.3)	
Mixed cell melanoma	105 (7.4)	63 (6.3)	42 (9.8)	
**T stage**				0.850
T1-T2	1124 (78.8)	789 (78.9)	335 (78.5)	
T3-T4	303 (21.2)	211 (21.1)	92 (21.5)	
**N stage**				0.895
N0	1398 (98.0)	980 (98.0)	418 (97.9)	
N1	29 (2.0)	20 (2.0)	9 (2.1)	
**M stage**				0.874
M0	1408 (98.7)	987 (98.7)	421 (98.6)	
M1	19 (1.3)	13 (1.3)	6 (1.4)	
**Surgery**				0.773
No	1060 (74.3)	745 (74.5)	315 (73.8)	
Yes	367 (25.7)	255 (25.5)	112 (26.2)	
**Radiotherapy**				0.032
No	335 (23.5)	219 (21.9)	116 (27.2)	
Yes	1092 (76.5)	781 (78.1)	311 (72.8)	
**Chemotherapy recode**				0.506
No	1405 (98.5)	986 (98.6)	419 (98.1)	
Yes	22 (1.5)	14 (1.4)	8 (1.9)	
**Stage**				0.702
Localized	1297 (90.9)	912 (91.2)	385(90.2)	
Regional	108 (7.6)	72 (7.2)	36 (8.4)	
Distant	22 (1.5)	16 (1.6)	6 (1.4)	

### Independent Predictors for Overall Survival of Elderly Patients With Uveal Melanoma

The univariate Cox analysis suggested that age, marital status, sex, primary site, histologic type, T stage, N stage, M stage, surgery, RT, and historic stage were statistically significant OS-related variables (all *p*-values <0.05) (Table 2). All these variables were then incorporated into the multivariate Cox analysis; age, marital status, sex, T stage, N stage, M stage, and RT were finally identified as the independent prognostic factors for elderly patients with UM (Table 2).

**TABLE 2:** Univariate and multivariate Cox regression analysis for elderly patients with UM.

Variables	Univariate analysis		Multivariate analysis	
	HR (95%CI) *P-*value		HR (95%CI) *P-*value	
**Age**				
60–69	Reference			
70–82	1.682 (1.309–2.161)	<0.001	1.663 (1.290–2.144)	<0.001
83–98	3.727 (2.695–5.154)	<0.001	3.681 (2.600–5.212)	<0.001
**Marital status**				
Married	Reference			
Unmarried	1.422 (1.110–1.822)	0.005	1.418 (1.108–1.814)	0.006
**Insurance status**				
Insured	Reference			
Uninsured	1.854 (0.691–4.977)	0.220		
**Sex**				
Female	Reference			
Male	1.281 (1.022–1.606)	0.032	1.435 (1.126–1.829)	0.004
**Race**				
Black	Reference			
Other	2.226 (0.462–10.723)	0.318		
White	1.060 (0.264–4.262)	0.935		
**Primary site**				
Choroid	Reference			
Ciliary body	1.384 (1.008–1.900)	0.045		
**Laterality**				
Left	Reference			
Right	1.004 (0.802–1.257)	0.972		
**Histologic type**				
Malignant melanoma	Reference			
Spindle cell melanoma	0.491 (0.275–0.876)	0.016		
Spithelioid cell melanoma	1.916 (1.187–3.092)	0.008		
Mixed cell melanoma	1.776(1.201–2.626)	0.004		
**T stage**				
T1-T2	Reference			
T3-T4	2.351 (1.840–3.003)	<0.001	2.270 (1.752–2.941)	<0.001
**N stage**				
N0	Reference			
N1	4.320 (2.712–6.880)	<0.001	2.233 (1.379–3.617)	0.001
**M stage**				
M0	Reference			
M1	3.591 (1.847–6.981)	<0.001	4.024 (2.031–7.971)	<0.001
**Surgery**				
No	Reference			
Yes	1.640 (1.291–2.082)	<0.001		
**Radiotherapy**				
No	Reference			
Yes	0.509 (0.399–0.649)	<0.001	0.690 (0.535–0.890)	0.004
**Chemotherapy**				
No/Unknown	Reference			
Yes	1.708 (0.761–3.834)	0.195		
**Stage**				
Localized	Reference			
Regional	1.401 (0.960–2.045)	0.080		
Distant	3.795 (2.123–6.785)	<0.001		

### Construction and Validation of the Nomogram

**FIGURE 2:**
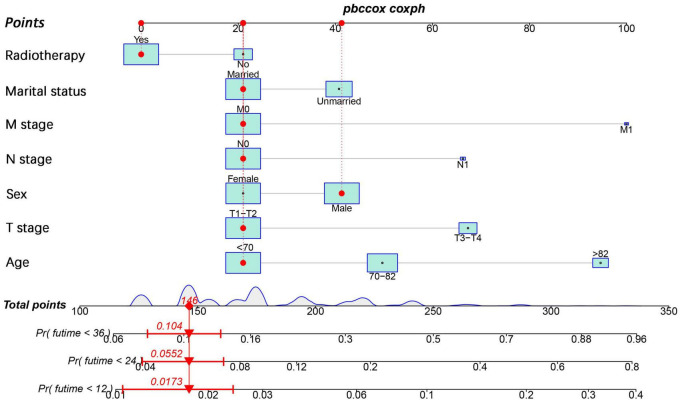
Prognostic nomogram for predicting 1-, 2- and 3-year OS of elderly patients with UM.

**FIGURE 3:**
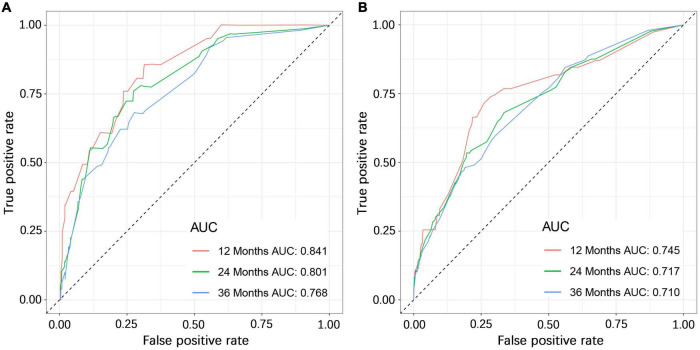
The ROC (receiver operating characteristic) curves of the training cohort **(A)** and validation cohort **(B)**.

**FIGURE 4:**
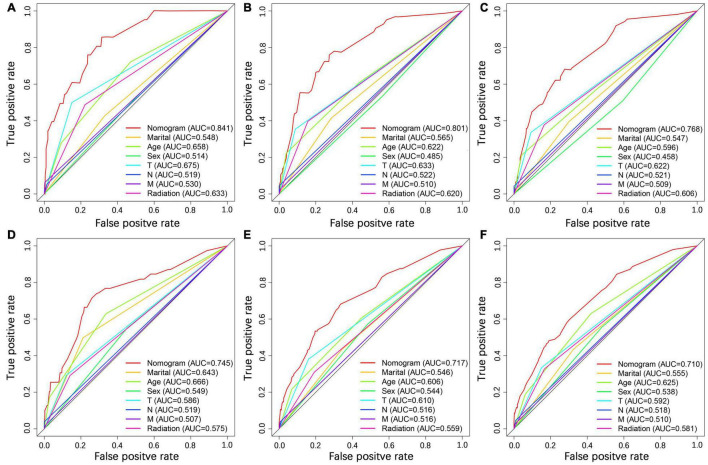
Comparison of ROC curves between the nomogram and independent predictors at 1 **(A)**, 2 **(B)**, and 3 years **(C)** in the training cohort and at 1 **(D)**, 2 **(E)**, and 3 year **(F**) in the validation cohort.

**FIGURE 5:**
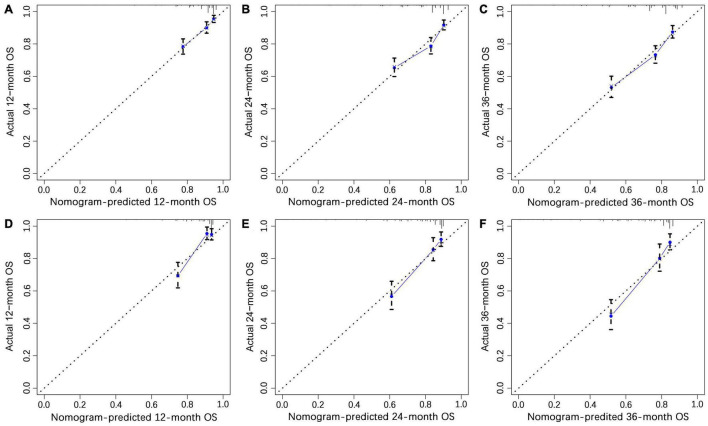
The calibration curves of 1- **(A)**, 2- **(B)**, and 3-year **(C)** overall survival (OS) in the training cohort and 1- **(D)**, 2- **(E)**, and 3-year **(F)** OS in the validation cohort.

**FIGURE 6:**
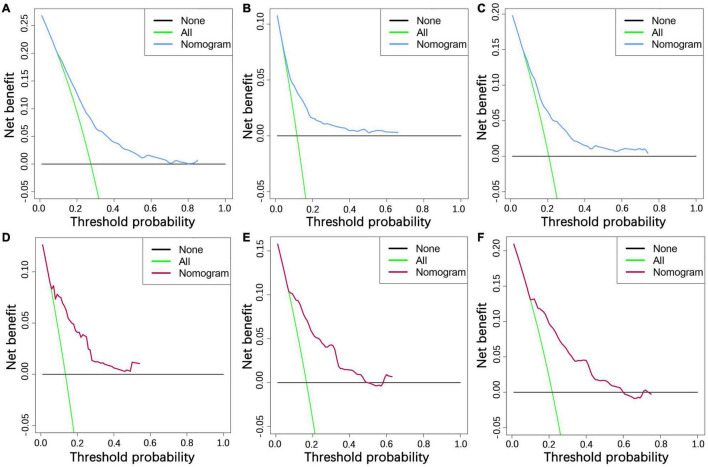
The decision curve analysis of 1- **(A)**, 2- **(B)**, and 3-year **(C)** OS in the training cohort and 1- **(D)**, 2- **(E)**, and 3-year **(F)** OS in the validation cohort.

We constructed a nomogram to predict the 1-, 2-, and 3-year OS of elderly patients with UM based on the abovementioned factors (Figure 2). Based on this model, we could select the subcategories of each predictor according to individual characteristics and get specific points by drawing a vertical line to the point axis at the upper end. Then, the total points could be calculated by adding up the points of all predictors together to estimate the 1-, 2-, and 3-year survival probability. Furthermore, the nomogram was validated in the training cohort and the validation cohort. In the training cohort, the AUC values of the nomogram predicting the 1-, 2-, and 3-year OS were 0.841, 0.801, and 0.768, respectively. In the validation cohort, the AUC values of the nomogram predicting the 1-, 2-, and 3-year OS were 0.745, 0.717, and 0.710, respectively (Figure 3). We further compared the difference of predictive performance between each independent predictor and the comprehensive model and found that the AUC of the nomogram was higher than the AUCs of all independent predictors at different time points (Figure 4). The calibration curves showed an optimal agreement between 1-, 2-, and 3-year predictions by nomogram and an actual observation in the two cohorts (Figure 5). As shown in Figure 6, the nomogram showed great positive net benefits across wide ranges of death risk in both cohorts, indicating its favorable clinical utility in predicting 1-, 2-, and 3-year OS.

**FIGURE 7:**
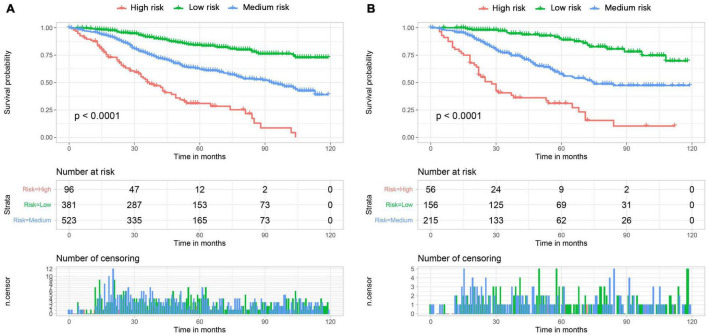
The Kaplan-Meier survival curves of three mortality risk subgroups in the training cohort **(A)** and validation cohort **(B)**.

Besides, based on optimal cutoff values obtained using X-tile software, patients in the two cohorts were classified into three subgroups, each of which contained patients with different risk of death, including low-risk group (score <166), medium-risk group (score 166–233), or high-risk group (score >233). The Kaplan–Meier survival curves showed that patients who were assigned to the high-risk group had a worst survival outcome in both cohorts (*p* < 0.05) (Figure 7). Patients with poorer prognosis (those who are at high risk of death) tend to have clinical characteristics of older age, no RT, unmarried status, men, and advanced TNM stage.

### Development of a Dynamic Web-Based Calculator for This Nomogram

**FIGURE 8:**
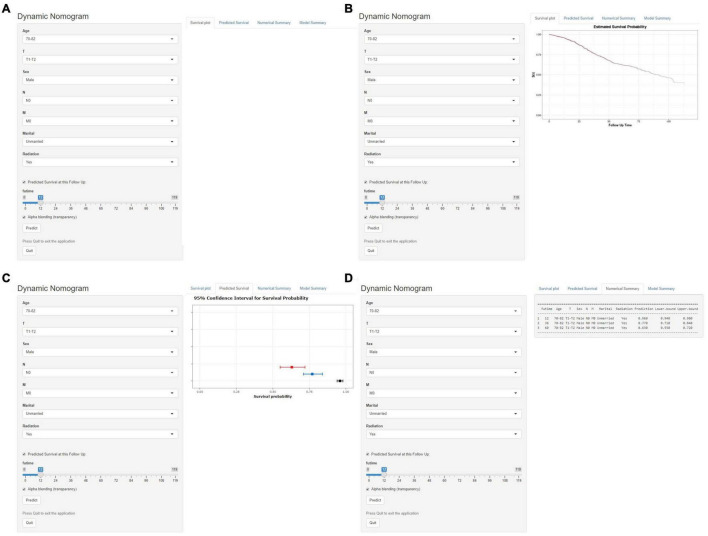
Operation interface of web-based calculator. After inputting a patient’ s age, sex, T stage, N stage, M stage, marital status, radiotherapy status on the web, and ascertaining time point of survival prediction **(A)**. Then, survival probability **(B)**, graphical summary predicting 1-year (black), 3-year (blue), and 5-year (red) OS and 95% CIs **(C)**, and a numerical summary showing the actual values of probability and 95% CIs **(D)** will be available. OS, overall survival; CI, confidence interval.

Based on the model, we developed a dynamic web-based calculator to simplify the application of this nomogram, which can be accessible *via* https://yuexinupup.shinyapps.io/DynNomapp/ (Figure 8). It is convenient to predict survival probability and its 95% confidence interval (CI) by inputting their clinical features. For instance, for an unmarried 76-year-old male patient with T1, N0, and M0, after receiving radiation, the 5-year OS rate was approximately 62.0% (95% CI, 54.0–72.0).

## Discussion

The UM may occur at any age, yet it is quite rare among children and young adults (16). The incidence increases with age, reaching a constant level at the age of 75 years, whereas the average age at the diagnosis of this cancer ranges from 59 to 62 years (17, 18). Despite the efforts regarding prognostic assessment of patients with UM made by a previous study (6), the complexity and high incidence of elderly patients make their targeted management and further research particularly important. We performed a current retrospective analysis using data from the SEER program. This study was the first to develop a well-validated nomogram for elderly patients with UM. By obtaining the data of several easily accessible variables on the nomogram of each individual, the total score can be easily calculated. Later, the 1-, 2-, and 3-year OS for elderly patients with UM can be easily estimated on the nomogram, which can provide guidance for further clinical management. Furthermore, to make it more flexible in practical application, the web-based survival calculator was further developed. It can produce a target patient’s survival rate at a particular time point by inputting the value of each independent prognostic factor with a single mouse click, thereby improving the model efficiency dramatically.

In our study, seven easily accessible variables were identified as independent prognostic factors, including age, sex, marital status, T stage, N stage, M stage, and RT. Similar to the previous report, age was also confirmed as an independent prognostic factor (19). The result indicated that patients in the age group of >82 years faced the worst OS (HR: 3.681, 95% CI 2.600–5.212) and patients in the age group of <70 years enjoyed the most satisfying survival outcome. This finding is in accordance with the previous study conducted by Krantz B (3). One possible explanation is that elderly patients were often associated with decreased immune function, and they may have multiple comorbidities that affect the choice of treatment and tolerance to treatment. As a result, the challenge of reducing the burden of disease attributable to UM is enormous among this population.

In addition, it was found that marital status could significantly influence the survival outcome of elderly patients with UM. Loya et al. reported that harmonious marriage had a positive effect on patients with ocular and periocular malignancies (20). Common symptoms of UM included blurred vision and proptosis; without timely treatment, it can make the eye blind, inflamed, and painful. Such symptoms can attract the early attention of partners or spouses, who may urge patients to receive timely diagnosis and treatment. Besides, those patients who are married tend to have more powerful financial resources and more psychological support (21). It has also been documented that patients who are married display less depression and anxiety than those who are unmarried after the diagnosis of UM, since a partner can share the emotional burden and provide appropriate social support (22). Therefore, clinical doctors are supposed to pay more attention to assessing and relieving the psychological stress of unmarried elderly patients with UM as well as maximizing their treatment adherence. It has been reported that male patients generally have poorer survival than female patients in various types of cancer (23). A similar situation is also presented in our study; among these patients, the mortality risk is higher for male patients than for female patients; this may be attributable to behavioral habits differences and intrinsic changes following aging. Besides, men are more attracted by tobacco and alcohol than women. The behaviors made pathophysiological characteristics in the two sexes significantly change. In addition, the intrinsic sex hormones modulating immune responses, oxidative regulation, and DNA repair played an important role in cellular activities (24). Nevertheless, the detailed mechanism of the phenomenon has not been clearly established, and further investigation was needed. The AJCC/TNM staging system on the basis of the invasion extent of primary tumor (T stage), lymph node status (N stage), and distant spread (M stage) was widely accepted to differentiate patients in the clinical working environment. In this study, T stage, N stage, and M stage were also determined as independent prognostic factors for OS in elderly patients with UM. In contrast, differing points between the results of this study and the traditional AJCC/TNM staging system may lie in visualization and quantification of the extent to which different variables contribute to the prognosis, which is valuable for precision medicine. An interesting point to note is that the greatest risk of death had been exhibited by the patients with distant metastases at the initial diagnosis. It is almost an established fact that the presence of metastatic disease is the leading cause of death in patients with UM; patients experience an increased mortality rate in parallel with the occurrence of metastasis, and this challenge will be much tougher among the elderly population with declining physical function. It is, therefore, important to evaluate the distant metastases in patients with a primary diagnosis of UM. Several reports have indicated that in UM, the risk of metastases increases with increased tumor pigmentation (25, 26). Moreover, some potential non-invasive biomarkers in sera could be used to predict the metastasis and prognosis of melanoma, and miR-23a has been reported to suppress the expression of raf kinase inhibitor protein (RKIP), which was a metastasis inhibitor and inversely correlated with metastases in UM (27–29).

However, despite increasing knowledge in the biology of UM, the therapeutic strategy is still disputed. This study found that only RT can significantly improve the survival rate of elderly patients with UM. In the 1970s, enucleation was considered the only therapy option in the treatment of UM. It is currently reserved for cases with the worst visual prognosis, such as patients with large UM (tumoral thickness greater than 8 mm), with choroidal melanoma surrounding the optic nerve, or presenting with severe hemorrhage, retinal detachment, or vitreous hemorrhage (30). Zimmerman et al. reported that the patients undergoing surgery had worse clinical outcome than those undergoing RT treatment, which may be related to distant metastasis caused by the dissemination of the tumor emboli due to the surgery invasiveness (31). Nowadays, as a form of localized RT, plaque brachytherapy has become the most popular method and an important treatment modality for the majority of UM. It was reported that the treatment mainly depended on a radioactive implant which could provide an apex RT dose of 80–100 Gy by inserting into the episcleral tissue (32, 33). The study further showed the significant effects of RT on elderly patients with UM, instead of surgery. This may also be partly associated with the higher risk of surgery among elderly patients.

There are still several limitations to this study. First, it is difficult to avoid selection bias because this study was designed in a retrospective way. Second, there is a lack of external data from different regions due to the rarity of UM; therefore, further validation with external data is needed to verify whether these results are generally applicable. Third, we are unable to consider other clinical factors and biomarkers not collected in the database that may have affected the outcomes, such as target therapy, postoperative complications, gene expression, and chromosomal alteration.

## Conclusion

This study determined that age, sex, marital status, T stage, N stage, M stage, and RT were independent predictors for OS of elderly patients with UM. A nomogram and a web-based survival calculator were developed to distinguish the high-risk elderly patients with UM, which might help ophthalmologist to develop better clinical management and treatment strategies.

## Data Availability Statement

The raw data supporting the conclusions of this article will be made available by the authors, without undue reservation.

## Author Contributions

ML and YT conceived and designed the study and critically reviewed the manuscript. ML performed the literature search, analyzed the data, and wrote the manuscript. ML and XY generated the figures and tables. YT supervised the research. All authors critically read the manuscript to improve intellectual content and read and approved the final manuscript.

## Conflict of Interest

The authors declare that the research was conducted in the absence of any commercial or financial relationships that could be construed as a potential conflict of interest.

## Publisher’s Note

All claims expressed in this article are solely those of the authors and do not necessarily represent those of their affiliated organizations, or those of the publisher, the editors and the reviewers. Any product that may be evaluated in this article, or claim that may be made by its manufacturer, is not guaranteed or endorsed by the publisher.

## Abbreviations

UM: Uveal melanoma
SEER: Surveillance, Epidemiology, and End Results
ROC: Receiver operating characteristic
DCA: Decision curve analysis
OS: Overall survival
AUC: Area under the curve
AJCC: American Joint Committee on Cancer
CI: Confidence interval
HR: Hazard ratio.

## References

[1] Aronow M Topham A Singh AD. Uveal melanoma: 5-year update on incidence, treatment, and survival (SEER 1973-2013). *Ocul Oncol Pathol.* (2018) 4:145–51. 10.1159/000480640 29765944 PMC5939716

[2] Kaliki S Shields CJE. Uveal melanoma: relatively rare but deadly cancer. *Eye (Lond).* (2017) 31:241–57. 10.1038/eye.2016.275 27911450 PMC5306463

[3] Krantz B Dave N Komatsubara K Marr B Carvajal RD. Uveal melanoma: epidemiology, etiology, and treatment of primary disease. *Clin Ophthalmol.* (2017) 11:279–89. 10.2147/OPTH.S89591 28203054 PMC5298817

[4] Danilova N Davydova SY. Immunohistochemical characteristics of uveal melanoma assording to the age at diagnosis, histological type and extension of the tumor. *Arkh Patol.* (2014) 76:55–60. 25543409

[5] Shields C Kaliki S Arepalli S Atalay HT Manjandavida FP Pieretti G Uveal melanoma in children and teenagers. *Saudi J Ophthalmol.* (2013) 27:197–201. 10.1016/j.sjopt.2013.06.013 24227986 PMC3770213

[6] Zeng Q Yao Y Zhao M. Development and validation of a nomogram to predict cancer-specific survival of uveal melanoma. *BMC Ophthalmol.* (2021) 21:230. 10.1186/s12886-021-01968-6 34030647 PMC8147099

[7] Dika E Lambertini M Pellegrini C Veronesi G Melotti B Riefolo M Cutaneous and mucosal melanomas of uncommon sites: where do we stand now? *J Clin Med.* (2021) 10:478. 10.3390/jcm10030478 33525348 PMC7866093

[8] Lambertini M Patrizi A Fanti P Melotti B Caliceti U Magnoni C Oral melanoma and other pigmentations: when to biopsy? *J Eur Acad Dermatol Venereol.* (2018) 32:209–14. 10.1111/jdv.14574 28862771

[9] Kaliki S Shields C Shields JA. Uveal melanoma: estimating prognosis. *Indian J Ophthalmol.* (2015) 63:93–102. 10.4103/0301-4738.154367 25827538 PMC4399142

[10] Shah C Weis E Lajous M Shields J Shields CL. Intermittent and chronic ultraviolet light exposure and uveal melanoma: a meta-analysis. *Ophthalmology.* (2005) 112:1599–607. 10.1016/j.ophtha.2005.04.020 16051363

[11] Regan S Judge H Gragoudas E Egan KM. Iris color as a prognostic factor in ocular melanoma. *Arch Ophthalmol.* (1999) 117:811–4. 10.1001/archopht.117.6.811 10369595

[12] Spagnolo F Caltabiano G Queirolo P. Uveal melanoma. *Cancer Treat Rev.* (2012) 38:549–53. 22270078 10.1016/j.ctrv.2012.01.002

[13] Nichols E Richmond A Daniels AB. Tumor characteristics, genetics, management, and the risk of metastasis in uveal melanoma. *Semin Ophthalmol.* (2016) 31:304–9. 10.3109/08820538.2016.1154175 27128983 PMC5526754

[14] Balachandran VP Gonen M Smith JJ Dematteo RP. Nomograms in oncology: more than meets the eye. *Lancet Oncol.* (2015) 16:e173–80. 10.1016/S1470-2045(14)71116-7 25846097 PMC4465353

[15] Camp R Dolled-Filhart M Rimm DL. X-tile: a new bio-informatics tool for biomarker assessment and outcome-based cut-point optimization. *Clin Cancer Res.* (2004) 10:7252–9. 10.1158/1078-0432.CCR-04-0713 15534099

[16] Al-Jamal R Cassoux N Desjardins L Damato B Konstantinidis L Coupland S The pediatric choroidal and ciliary body melanoma study: a survey by the European ophthalmic oncology group. *Ophthalmology.* (2016) 123:898–907. 10.1016/j.ophtha.2015.12.024 26854035

[17] Shields C Furuta M Thangappan A Nagori S Mashayekhi A Lally D Metastasis of uveal melanoma millimeter-by-millimeter in 8033 consecutive eyes. *Arch Ophthalmol.* (2009) 127:989–98. 10.1001/archophthalmol.2009.208 19667335

[18] Singh A Turell M Topham AJO. Uveal melanoma: trends in incidence, treatment, and survival. *Ophthalmology.* (2011) 118:1881–5. 10.1016/j.ophtha.2011.01.040 21704381

[19] Fallico M Raciti G Longo A Reibaldi M Bonfiglio V Russo A Current molecular and clinical insights into uveal melanoma (Review). *Int J Oncol.* (2021) 58:10. 10.3892/ijo.2021.5190 33649778 PMC7910016

[20] Loya A Ayaz T. Weng CJCo: impact of marital status on survival in patients with ocular and periocular malignancies: a retrospective analysis of 3159 patients from the SEER database. *Clin Ophthalmol.* (2020) 14:1127–33. 10.2147/OPTH.S238034 32425498 PMC7186878

[21] Aizer A Chen M McCarthy E Mendu M Koo S Wilhite T Marital status and survival in patients with cancer. *J Clin Oncol.* (2013) 31:3869–76. 24062405 10.1200/JCO.2013.49.6489 PMC4878087

[22] Reiche E Nunes S Morimoto HK. Stress, depression, the immune system, and cancer. *Lancet Oncol.* (2004) 5:617–25. 10.1016/S1470-2045(04)01597-9 15465465

[23] Kim H Lim H Moon AJB. Sex differences in cancer: epidemiology, Genetics and Therapy. *Biomol Ther (Seoul).* (2018) 26:335–42. 10.4062/biomolther.2018.103 29949843 PMC6029678

[24] Mitkov M Joseph R Copland JIII. Steroid hormone influence on melanomagenesis. *Mol Cell Endocrinol.* (2015) 417:94–102. 10.1016/j.mce.2015.09.020 26415591

[25] Shields C Kaliki S Cohen M Shields P Furuta M Shields JJE. Prognosis of uveal melanoma based on race in 8100 patients: the 2015 doyne lecture. *Eye (Lond).* (2015) 29:1027–35. 10.1038/eye.2015.51 26248525 PMC4541345

[26] Slominski A Kim T Brożyna A Janjetovic Z Brooks DLP Schwab L Seagroves TJAob, biophysics: the role of melanogenesis in regulation of melanoma behavior: melanogenesis leads to stimulation of HIF-1α expression and HIF-dependent attendant pathways. *Arch Biochem Biophys.* (2014) 563:79–93. 10.1016/j.abb.2014.06.030 24997364 PMC4221528

[27] Hatzl S Geiger O Kuepper M Caraffini V Seime T Furlan T Increased expression of miR-23a mediates a loss of expression in the RAF kinase inhibitor protein RKIP. *Cancer Res.* (2016) 76:3644–54. 10.1158/0008-5472.CAN-15-3049 27197200 PMC5024755

[28] Das S Bhutia S Sokhi U Azab B Su Z Boukerche H Raf kinase inhibitor RKIP inhibits MDA-9/syntenin-mediated metastasis in melanoma. *Cancer Res.* (2012) 72:6217–26. 10.1158/0008-5472.CAN-12-0402 23066033 PMC3939082

[29] Caltabiano R Puzzo L Barresi V Cardile V Loreto C Ragusa M Expression of raf kinase inhibitor protein (RKIP) is a predictor of uveal melanoma metastasis. *Histol Histopathol.* (2014) 29:1325–34. 10.14670/HH-29.1325 24763848

[30] Vivet-Noguer R Tarin M Roman-Roman S. Alsafadi. emerging therapeutic opportunities based on current knowledge of uveal melanoma biology. *Cancers.* (2019) 11:1019. 10.3390/cancers11071019 31330784 PMC6678734

[31] Zimmerman L McLean I Foster WD. Does enucleation of the eye containing a malignant melanoma prevent or accelerate the dissemination of tumour cells. *Br J Ophthalmol.* (1978) 62:420–5. 10.1136/bjo.62.6.420 352389 PMC1043246

[32] Sandinha M Farquharson M McKay I Roberts F. Monosomy 3 predicts death but not time until death in choroidal melanoma. *Invest Ophthalmol Vis Sci.* (2005) 46:3497–501. 10.1167/iovs.05-0613 16186325

[33] Shields C Kaliki S Furuta M Fulco E Alarcon C Shields JA. American joint committee on cancer classification of posterior uveal melanoma (tumor size category) predicts prognosis in 7731 patients. *Ophthalmology.* (2013) 120:2066–71. 10.1016/j.ophtha.2013.03.012 23664467

